# The Institutes of Medicine

**Published:** 1860-09

**Authors:** 


ART. XIII.—*The Institutes of Medicine.* By MARTYN PAINE, A.M., M.D., LL.D., Professor of the Institutes of Medicine and Materia Medica in the University of the City of New York, etc. etc. Fifth edition; 8vo., pp. 1109. New York: Harper & Brothers, 1859.

THAT five editions of this philosophical and deservedly popular work should have been required within the space of fifteen years is sufficient evidence of the high estimate in which it is held by the profession. In the September issue, 1858, of the REVIEW, we presented our readers with an elaborate notice of Dr. Paine's work, and the present edition does not materially differ from the former. The appendix has undergone some modifications, and a supplement has been added, which contains observations on the correlation of forces, the glycogenic function of the liver, the cause of the blood's fluidity, the *modus operandi* of medicines, absorption by the skin, transfusion of remedies, intestinal absorption and lacteal circulation, and the forces which circulate the blood. Although the author's views are peculiar in many respects, and not sustained by the majority of physiologists of the present day, yet no one can fail to wonder at the great storehouse of erudition displayed in this volume, which should be in the hands of every accomplished physician throughout the land.

